# Dropout rates and factors associated with adherence to opioid agonist treatment among adults with opioid use disorder at Sekou Toure Regional Referral Hospital, Mwanza, Tanzania

**DOI:** 10.3389/fpsyt.2026.1660196

**Published:** 2026-03-04

**Authors:** Tusherahma Hussein Tuli, Allen Rweyendera, Greyson Gwahula, Yacinter Vedastus, Raymond Maziku, Peter Chilipweli, Kiyeti A. Hauli

**Affiliations:** 1 School of Medicine, Catholic University of Health and Allied Sciences (CUHAS), Mwanza, Tanzania; 2 Development of Research and Consultancy, Community Health and Development Foundation (CHADF), Mwanza, Tanzania; 3 Department of Community Medicine, School of Public Health, Catholic University of Health and Allied Sciences (CUHAS), Mwanza, Tanzania; 4 Department of Psychiatry, Bugando Medical Center and Catholic University of Health and Allied Sciences (CUHAS), Mwanza, Tanzania

**Keywords:** adherence, dropout, heroin use, methadone, opioid agonist treatment, Tanzania

## Abstract

**Background:**

Opioid use remains a significant global public health concern, with approximately 60 million people using opioids and 39.5 million living with opioid use disorders. Opioid agonist treatment (OAT), particularly methadone, is an effective intervention for opioid dependence, though retention remains problematic worldwide. This study aimed to determine the dropout rate and identify factors influencing adherence to OAT among patients at Sekou Toure Regional Referral Hospital (SRRH) in Mwanza, Tanzania.

**Methods:**

A mixed-method design combining a retrospective cohort and cross-sectional approach was used. Retrospective data were extracted from records of patients enrolled from February 2018 to March 2024. A cross-sectional survey was conducted from September to November 2024 among 223 systematically selected patients. Data analysis included descriptive statistics and Chi-square tests, with significance set at p < 0.05.

**Results:**

The dropout rate was 37.9%. Most dropouts were men (97.4%), consistent with the predominantly male patient population. Nearly half of the dropouts (47.7%) had only primary education. Adherence was influenced by personal motivations such as improving health (27.8%) and maintaining sobriety (27.4%), social support (25.6% reported none), and psychological distress (38.1% reported severe symptoms). Perceived treatment effectiveness varied, with 46.6% rating OAT as effective or very effective.

**Conclusion:**

The high dropout rate highlights the need for targeted interventions focused on men, individuals with lower education levels, and patients lacking social support. Strengthening the treatment environment, enhancing psychological support, and improving communication about OAT effectiveness may improve retention. Further research is needed to understand barriers to confidence in OAT and perceptions of treatment ineffectiveness.

## 1 Introduction

Opioid use remains a major global public health challenge. According to the 2023 World Drug Report, 296 million people used drugs in 2021, with amphetamine-type stimulants being more widely used globally than opioids. Approximately 60 million people used opioids, and 31.5 million used opiates such as heroin. Opioids contribute to the majority of drug-related deaths, mainly due to overdose, with risk highest shortly after treatment discontinuation when tolerance has diminished (1).

Opioid agonist treatment (OAT), including methadone, buprenorphine, slow-release oral morphine (SROM), and heroin-assisted treatment (HAT), is the most effective intervention for opioid dependence. These medications alleviate withdrawal symptoms, reduce cravings, and decrease the likelihood of relapse, overdose, and infectious disease transmission. Antagonist medications such as naltrexone play a more limited role globally due to adherence barriers and restricted availability in low-resource settings.

In Tanzania, OAT was introduced in 2011 and has expanded to multiple regions, including Mwanza. Despite this progress, retention remains a major challenge. Studies from Dar es Salaam have documented dropout rates ranging from 30% to 40% (2, 3), with retention influenced by methadone dosing, psychosocial support, comorbid mental health conditions, stigma, transport challenges, and facility-level factors.

However, most evidence comes from Dar es Salaam, and little is known about adherence and dropout patterns in regional referral hospitals such as SRRH in Mwanza. This represents a critical knowledge gap, particularly because treatment discontinuation is associated with relapse, overdose, increased mortality, and poor social outcomes.

Therefore, this study aimed to determine the dropout rate and identify factors influencing adherence to OAT among patients attending Sekou Toure Regional Referral Hospital in Mwanza, Tanzania.

## 2 Methodology

### 2.1 Study area

The study was conducted at the Methadone Clinic of Sekou Toure Regional Referral Hospital (SRRH) in Mwanza, Tanzania. The hospital serves approximately 2.2 million people in the Lake Zone and provides methadone-based OAT to a large population of adults with opioid use disorder.

### 2.2 Study design

A mixed-methods approach combining a retrospective cohort and cross-sectional design was used. The retrospective component assessed OAT dropout using clinical records of patients enrolled between February 2018 and March 2024. The cross-sectional survey, conducted from September to November 2024, assessed current factors associated with adherence among active patients. This integration allowed examination of both historical trends and current determinants of adherence.

### 2.3 Study population

The study population included all heroin-dependent patients enrolled in OAT between February 2018 and March 2024 and who had been on treatment for at least six months. Complete clinic records up to March 2024 were available. Files with significant missing information were excluded to ensure data validity.

### 2.4 Sample size estimation, selection criteria and sampling method

Sample size was calculated using the Kish–Leslie formula with a 95% confidence interval, 5% margin of error, and a prevalence of heroin use (17.6%) from a national Tanzanian survey. Because no reliable dropout prevalence data existed for Mwanza at the time of planning, heroin-use prevalence served as the best available estimate.

“Heroin smoking alone” refers to individuals who used heroin exclusively by smoking rather than injection.

A systematic random sampling technique was used to select 223 eligible patients.

Inclusion criteria:

- Verified heroin use confirmed through clinical assessment, patient history, and urine drug screening
- Enrollment in OAT for ≥6 months
- Attendance at SRRH during the study period

Exclusion criteria:

Primary dependence on non-opioid substances.

Patients with co-occurring substance use were included if heroin was the primary drug.

### 2.5 Data collection data analysis

Data were collected using two tools: a checklist and a self-administered questionnaire. The checklist was used to extract secondary data from clinic records regarding patients’ dropout rates from methadone-assisted treatment (OAT), while the self-administered questionnaire was designed to assess factors contributing to adherence among patients receiving OAT. The questionnaire captured variables such as socio-demographic characteristics, duration on treatment, support systems, and individual perceptions toward OAT. All completed questionnaires were coded and assigned serial numbers for ease of reference and data management. The collected data were compiled, cleaned, and analyzed using the Statistical Package for the Social Sciences (SPSS) software version 26. Descriptive statistics including frequencies and percentages were used to summarize categorical variables. Cross-tabulation was performed to explore associations between independent variables and adherence status, and Chi-square tests were used to determine statistical significance. A p-value of less than 0.05 was considered statistically significant.

### 2.6 Ethics

Ethical approval was soughed from the Joint BMC/CUHAS Research Ethical Committee and Director of research and innovation CUHAS numbered CRECU/3378/2024. Written informed consent was sought and obtained before the recruitment of study respondents, after they were provided with sufficient information about the risks and benefits of the study. Confidentiality was ensured, and those who agreed to participate signed the consent form, while illiterate participants provided a thumbprint.

## 3 Results

### 3.1 Sociodemographic information of study participants

This section presents the demographic characteristics of the study participants, including gender, age, education level, marital status and employment status (**Table 1**).

**Table 1:** Sociodemographic characteristics of study participants.

Variable	Categories	Frequency	Percentage
Gender	Female	91	11.0
	Male	730	89.0
Age group	18-25	178	21.7
	26-35	129	15.7
	36-45	161	19.6
	46-55	198	24.1
	56+	155	18.9
Educational level	No formal education	113	13.7
	Primary	392	47.7
	Secondary	248	30.1
	Tertiary (collage/university)	68	8.5
Marital status	Single	414	50.4
	Married	242	29.4
	Divorced	165	20.1
Employment	Employment	323	39.3
	Unemployed	498	60.7

### 3.2 Characteristics of adhered clients

Characteristics of patients who remained in methadone treatment provide insight into factors contributing to adherence (**Table 2**).

**Table 2:** Social demographics and characteristics of patients who adhered to treatment.

Variable	Categories	Frequency	Percentage
Age	18-25	54	24.2
	26-35	34	15.2
	36-45	44	19.7
	46-55	54	24.2
	56+	37	16.6
Gender	Female	19	8.5
	Male	204	91.5
Employment status	Employed	50	22.4
	Retired	64	28.7
	Student	15	6.7
	Unemployed	94	42.1
Educational level	No formal education	58	26.0
	Primary	55	24.7
	Secondary	46	20.6
	Tertiary	64	28.7
Living situation	Alone	35	15.7
	Other	51	22.9
	Shelter	51	22.9
	With family	50	22.4
	With friends	36	16.1
Treatment period	6–12 months	52	23.3
	1–2 years	67	30.0
	2+ years	104	46.6

### 3.3 Dropout rate

**Figure 1:**
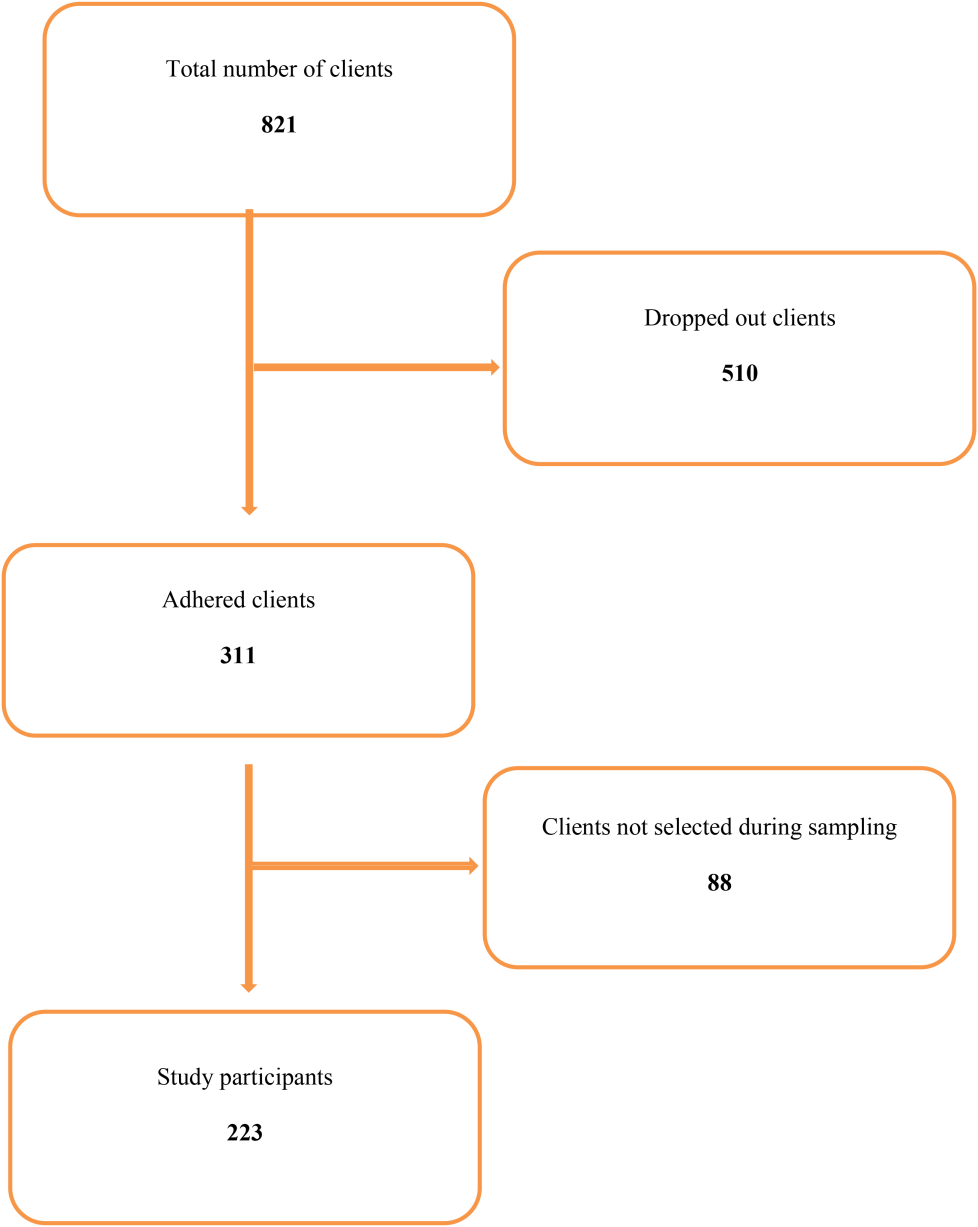
Flow diagram of study patients included in the analysis.

The dropout rate among patients enrolled in OAT at SRRH was 37.9%. Of the 821 patients registered between 2018 and 2024, 510 discontinued treatment. Most patients were male, and this distribution was mirrored among dropouts (97.4%). Therefore, the high proportion of male dropouts reflects the underlying gender distribution of the clinic population (**Figure 1**; **Table 3**).

**Table 3:** Demographics of the dropout patients.

Variable	Categories	Frequency	Percentage
Gender	Female	4	2.6
	Male	149	97.4
Educational level	No formal education	21	13.7
	Primary	73	47.7
	Secondary	46	30.1
	Tertiary (collage/university)	13	8.5
Marital status	Divorced	27	17.6
	Married	45	29.4
	Single	81	52.9

Dropout Rate (%)=Clients Continuing Service  Total clients registered ×100


Dropout Rate (%)=311 821 ×100=37.9

Dropout Rate (%)=37.9%

### 3.4 Factors for adherence to medication assisted treatment

Living situations further illustrate the patients’ varied support systems, with 22.9% each living in shelters or other arrangements, 22.4% living with family, 16.1% with friends, and 15.7% living alone. Treatment duration plays a crucial role in adherence, with the largest group (46.6%) having been on treatment for more than 2 years, followed by those on treatment for one to two years (26.9%), 6–12 months (23.3%). These findings underscore the importance of targeted interventions tailored to specific age groups, gender identities, and socioeconomic backgrounds to improve adherence rates and support systems among patients with heroin dependence undergoing OAT.

Factors influencing adherence to Opioid Agonist Treatment (OAT) among patients with heroin dependence at Sekou-Toure Methadone Clinic highlight the critical role of personal motivations and beliefs. Clients cited improving health (27.8%), maintaining sobriety (27.4%), reuniting with family (22.9%), and gaining employment (22.0%) as key personal goals for adherence. Confidence levels varied, with 26.5% feeling very confident in their ability to adhere to treatment, while 16.6% were very unconfident. Motivational factors included legal concerns (24.2%), family (21.5%), health (19.7%), personal growth (19.3%), and employment (15.2%). Perceived effectiveness of OAT also played a role, with 23.3% rating it as very effective and 23.3% as effective, compared to 20.6% who found it ineffective and 13.5% who deemed it very ineffective. These findings underline the importance of addressing patients’ personal goals and reinforcing confidence and perceived effectiveness to enhance treatment adherence.

Social support significantly influences adherence to Opioid Agonist Treatment (OAT) among patients with heroin dependence at Sekou-Toure Methadone Clinic. The strength of support networks varied, with 27.8% describing their support as moderate or weak, while 18.8% reported having strong support, and 25.6% indicated no support at all. Communication frequency with their support networks showed that 29.6% rarely communicated, 26.5% communicated weekly, 22.9% daily, and 21.1% monthly. Regarding provider support, 20.2% rated it as excellent and 18.8% as good, while 23.3% found it poor, 15.7% very poor, and 22.0% remained neutral. These findings underscore the need to strengthen both personal and provider-based support systems to improve treatment adherence.

Psychological factors play a crucial role in adherence to Opioid Agonist Treatment (OAT) among patients with heroin dependence at Sekou-Toure Methadone Clinic. A significant portion of patients (38.1%) reported experiencing severe psychological issues, while 29.1% reported mild issues, and 32.7% reported none. To manage cravings or urges to use heroin, 41.7% sought support from others, 35.4% engaged in other activities, 17.5% were unsure of their approach, and 5.4% used coping strategies. Counseling engagement was also notable, with 52.9% attending occasionally, 30.5% regularly, and 16.6% not engaging at all. These findings highlight the need for integrated psychological support and counseling services to enhance treatment adherence.

The treatment environment significantly affects adherence to Opioid Agonist Treatment (OAT) at Sekou-Toure Methadone Clinic. Regarding the health facility’s environment, 33.6% of patients found it neutral, 25.6% described it as supportive but needing improvement, 22.9% considered it unsupportive, and 17.9% viewed it as very supportive and welcoming. Privacy during treatment was another key factor, with 39.0% indicating they had adequate privacy sometimes, 23.8% stating they always had privacy, and 37.2% reporting a lack of privacy. These findings underscore the importance of creating a more welcoming and private treatment environment to foster greater adherence among patient (**Table 4**).

**Table 4:** Showing factors for adherence to methadone assisted treatment.

Variable	Categories	Frequency	Percentage
Personal motivations and beliefs			
Personal goals	Gain employment	49	22
	Improve health	62	27.8
	Maintain sobriety	61	27.4
	Reunite with family	51	22.9
Confidence in adherence	Confident	39	17.5
	Neutral	47	21.1
	Not confident	41	18.4
	Very confident	59	26.5
	Very unconfident	37	16.6
Motivation	Employment	34	15.2
	Family	48	21.5
	Health	44	19.7
	Legal	54	24.2
	Personal growth	43	19.3
Effectiveness	Effective	52	23.3
	Ineffective	46	20.6
	Neutral	43	19.3
	Very effective	52	23.3
	Very ineffective	30	13.5
Social support			
Support network	Moderate	62	27.8
	None	57	25.6
	Strong	42	18.8
	Weak	62	27.8
Communication frequency	Daily	51	22.9
	Monthly	47	21.1
	Rarely	66	29.6
	Weekly	59	26.5
Provider support	Excellent	45	20.2
	Good	42	18.8
	Neutral	49	22
	Poor	52	23.3
	Very poor	35	15.7
Psychological factors			
Experiencing severe Psychological issues	yes, severe	85	38.1
	Yes, mild	65	29.1
	no	73	32.7
Cravings or urges to use heroin	Engaging in other activities	79	35.4
	Not sure	39	17.5
	Seeking support from others	93	41.7
	Using coping strategies	12	5.4
Engaged in any counseling	Yes, occasionally	118	52.9
	Yes, regularly	68	30.5
	No	37	16.6
Treatment environment			
Environment of heath facility	Neutral	75	33.6
	Supportive but could improve	57	25.6
	Unsupportive	51	22.9
	Very supportive and welcoming	40	17.9
Adequate privacy	No	83	37.2
	Yes, always	53	23.8
	Yes, sometimes	87	39

### 3.5 Comparison of dropout and adhered participants

A comparison of dropout participants and those who adhered to methadone treatment based on similar characteristics (**Table 5**).

**Table 5:** Comparison of dropout and adhered patients.

Variable	Adhered (%)	Dropped out (%)	Chi-square	p-value
Gender				
Male	91.5	97.4	5.12	0.02
Female	8.5	2.6		
Age group				
18-25	24.2	21.7	1.12	0.45
26-35	15.3	15.7		
36-45	19.7	19.6		
46-55	24.2	24.1		
56+	16.6	18.9		
Education level				
- Primary	47.7	47.7	4.56	0.04
- Secondary (%)	30.1	30.1		
- Higher	13.7	8.5		
Employment status				
- Employed	22.4	30.0	3.89	0.05
- Unemployed	42.1	70.0		

## 4 Discussion

### 4.1 Dropout rate

The dropout rate of 37.9% observed in this study among patients with heroin dependence undergoing Opioid agonist treatment (OAT), at Sekou-Toure Regional Referral Hospital is a concerning finding, with a higher proportion of male patients discontinuing treatment. This is consistent with global trends indicating that male drug patients are generally more likely to drop out of treatment compared to females, possibly due to various factors such as social stigmas or greater impulsivity (4). Moreover, the dropout rate in this study aligns with findings from a study conducted in Tanzania, where dropout rates in OAT programs ranged from 30% to 40% (5). These results highlight the challenges faced by OAT programs in retaining participants, especially males, and underscore the need for more targeted strategies to engage this population.

The social demographics of the dropout patients in this study revealed that the majority were male (97.4%), and many had limited educational backgrounds, with 47.7% having only primary education. These findings are consistent with previous studies that indicate a higher dropout rate among individuals with lower educational attainment, as they may face more significant socio-economic challenges (6). Additionally, the marital status of dropouts showed that the majority were single (52.9%), which could suggest a lack of strong family support, which is often a key factor in the successful continuation of addiction treatment (7). These demographic factors emphasize the importance of tailoring OAT interventions to address the specific needs of individuals with lower education levels and those lacking strong familial support.

### 4.2 Factors for adherence to OAT

Personal motivations and beliefs emerged as significant factors influencing adherence to OAT. Clients in this study reported that their primary goals were to improve health, maintain sobriety, and reunite with family. These goals align with findings from studies in other regions, which show that health improvement and family reunification are common motivators for individuals seeking treatment for substance use disorders (8). Interestingly, a substantial proportion of patients (26.5%) reported being very confident in adhering to treatment, while 16.6% were very unconfident, suggesting that confidence in treatment outcomes plays a critical role in whether patients remain in treatment (9). The perceived effectiveness of OAT also varied, with 46.6% finding it effective or very effective, highlighting the need for continuous reinforcement of the benefits of OAT to maintain motivation.

Social support was another key factor impacting adherence. The majority of patients in this study reported moderate or weak support networks, with 25.6% indicating no support at all. Research has shown that social support is one of the strongest predictors of treatment adherence in substance use disorders (10). In this study, patients who had strong family or peer support were more likely to stay in treatment, consistent with findings from studies that emphasize the importance of a supportive environment for individuals in OAT (11). However, a significant number of patients experienced inadequate privacy, which could contribute to a lack of trust and further discourage adherence to treatment. A more private and confidential treatment environment could therefore be crucial in improving retention rates.

Finally, the treatment environment itself played a role in adherence. While 33.6% of patients viewed the facility as neutral, 25.6% felt that it was supportive but could be improved, and 22.9% described it as unsupportive. These findings are in line with research that highlights the importance of a welcoming and supportive treatment environment in ensuring the success of OAT programs (12–14). The absence of adequate privacy for 37.2% of patients is particularly concerning, as privacy is a significant factor in patients’ comfort and trust in the treatment process. Enhancing privacy and creating a more welcoming environment could potentially improve both retention and overall treatment outcomes for patients undergoing OAT at this facility.

### 4.3 Study limitation

While this study provides valuable insights into the dropout rates and adherence factors in methadone-assisted treatment, several limitations should be considered, such as;

Limited generalizability as the study was conducted at a single treatment center and the findings may not be fully generalized to other regions healthcare settings, Potential confounding factors as variables such as mental health status and co-occurring substance use disorders where not controlled for in-depth which may have influenced adherence rates and incomplete records as some participants records may have been missing affecting the comprehensiveness of the dataset.

### 4.4 Recommendations

To improve adherence and reduce dropout rates in OAT programs, it is recommended that interventions focus on enhancing social support networks, particularly for patients with limited family support or lower educational attainment. Tailored strategies should be implemented to address male patients’ specific needs, including increasing their confidence in treatment and emphasizing the effectiveness of OAT. Furthermore, the treatment environment should be improved by ensuring adequate privacy and a more supportive, welcoming atmosphere. Regular psychological counseling and stronger provider support could also play a crucial role in improving retention. Additionally, further research is necessary to explore the underlying causes of low confidence and the perceived ineffectiveness of OAT in this context.

### 4.5 Conclusion

The findings of this study highlight significant factors influencing adherence and dropout rates among patients with heroin dependence undergoing Opioid agonist treatment (OAT), at Sekou-Toure Regional Referral Hospital. A dropout rate of 37.9% was observed, with male patients and those with lower educational levels being more prone to discontinuing treatment. Personal motivations such as improving health and maintaining sobriety, along with social support, psychological factors, and treatment environment, were all found to significantly impact treatment adherence. These findings are consistent with global research, underscoring the complexity of factors contributing to both successful treatment retention and dropout in OAT programs.

## Data Availability

The raw data supporting the conclusions of this article will be made available by the authors, without undue reservation.
